# Whole-brain morphological alterations associated with trigeminal neuralgia

**DOI:** 10.1186/s10194-021-01308-5

**Published:** 2021-08-13

**Authors:** Jiajie Mo, Jianguo Zhang, Wenhan Hu, Fang Luo, Kai Zhang

**Affiliations:** 1 Department of Neurosurgery, Beijing Tiantan Hospital, Capital Medical University, No. 119 South 4th Ring West Road, Fengtai District, Beijing, China; 2 Department of Neurosurgery, Beijing Neurosurgical Institute, Capital Medical University, No. 119 South 4th Ring West Road, Fengtai District, 100070 Beijing, China; 3 Department of Pain Management, Beijing Tiantan Hospital, Capital Medical University, No. 119 South 4th Ring West Road, Fengtai District, 100070 Beijing, China

**Keywords:** Trigeminal neuralgia, Surface-based analysis, Multivariate analysis, Machine learning, Systematic review

## Abstract

**Background:**

Novel neuroimaging strategies have the potential to offer new insights into the mechanistic basis for trigeminal neuralgia (TN). The present study aims to conduct whole-brain morphometry analyses of TN patients and to assess the value of group-level neocortical and subcortical structural patterns as tools for diagnostic biomarker exploration.

**Methods:**

Cortical thickness, surface area, and myelin levels in the neocortex were measured via magnetic resonance imaging (MRI). The radial distance and the Jacobian determinant of the subcortex in 43 TN patients and 43 matched controls were compared. Pattern learning algorithms were employed to establish the utility of group-level MRI findings as tools for predicting TN. An additional 40 control patients with hemifacial spasms were then evaluated to assess algorithm sensitivity and specificity.

**Results:**

TN patients exhibited reductions in cortical indices in the anterior cingulate cortex (ACC), the midcingulate cortex (MCC), and the posterior cingulate cortex (PCC) relative to controls. They further presented with widespread subcortical volume reduction that was most evident in the putamen, the thalamus, the accumbens, the pallidum, and the hippocampus. Whole brain-level morphological alterations successfully enable automated TN diagnosis with high specificity (TN: 95.35 %; disease controls: 46.51 %).

**Conclusions:**

TN is associated with a distinctive whole-brain structural neuroimaging pattern, underscoring the value of machine learning as an approach to differentiating between morphological phenotypes, ultimately revealing the full spectrum of this disease and highlighting relevant diagnostic biomarkers.

**Supplementary Information:**

The online version contains supplementary material available at 10.1186/s10194-021-01308-5.

## Introduction

Trigeminal neuralgia (TN) is defined by paroxysmal unilateral facial pain in a well-defined territory, resulting in symptoms of sporadic, sudden, shock-like or burning facial pain that most commonly occurs in the V2 region, or less commonly in the V3 and V1 regions. TN is most frequently a result of trigeminal nerve root entry zone (REZ) compression by the local vasculature [1, 2]. Neuroimaging techniques, particularly magnetic resonance imaging (MRI), are essential for the etiological subclassification of primary or secondary TN, as well as for the detection of vascular contact and the prediction of the degree of root compression [3]. While not associated with elevated mortality rates, TN is linked to significant anxiety and quality of life reductions in affected individuals. Prolonged vascular compression can result in focal demyelination, leading to inappropriate neuronal activation or ephaptic cross-talk as a consequence of the pathological cross-activation of large and small afferent fibers. These processes, in turn, can lead to secondary changes which ultimately sensitize nociceptive neurons in the trigeminal nucleus and higher brain structures, leading to the symptoms of TN [4, 5].

MRI-derived metrics can be analyzed to explore interactions among brain structures and associated clinical manifestations, potentially offering insight into the pathological basis for a range of disease states. A number of morphological abnormalities have been linked to other chronic pain conditions including fibromyalgia, phantom pain, and chronic head pain [6–8]. Chronic TN previously has been linked to heterogeneous changes in the structure of these brain regions. For example, most studies have reported that TN patients exhibit a reduction in gray matter (GM) volume, although several articles have also reported increases in GM volume in other brain regions [5, 9, 10]. As idiopathic pain disorders are generally thought to be centrally mediated, neuroimaging has the potential to offer insight into the mechanistic basis for pain experienced by patients. Abnormalities in GM characteristics may be pain-driven, dynamic, and progressive [11], although previous analyses focusing on a single unique feature (thickness) were found to be inadequate as a means of capturing the entirety of the TN-related brain network. Subcortical structures can also play a critical role in modulating pain, and further study of TN-related neocortico-subcortical abnormalities is thus warranted. Multiple neuroimaging features have been shown to be conducive to understanding the more complex nature of the brain network. Multivariate analysis permits the simultaneous analysis of all input variables while accounting for their interactions and thereby yielding greater statistical power. Thus, the results of such analysis can be more readily interpreted as a neural network signature [12].

The present study was designed to (1) compare structural abnormalities in TN patients and healthy controls in order to detect specific TN-related neocortical and subcortical structures that may be associated with TN, and (2) construct a support vector machine (SVM) model to evaluate the ability of TN patient-derived quantitative imaging markers to differentiate between TN patients, healthy controls, and a disease control cohort of hemifacial spasm (HFS) patients.

## Methods

### Patient selection

For the present study, patients diagnosed with TN as per the Criteria of the International Headache Society (ICHD 3) [13] and patients with classical HFS with a positive lateral spread response were included. In both disease cohorts, patients were refractory to pharmacological treatment and underwent microvascular decompression via a retrosigmoid approach. All patients exhibited a likely locus of neurovascular compression on the ipsilateral root in preoperative MRI scans, as confirmed by the intraoperative observation of distinct vascular compression. Healthy controls were free of any history of neurological conditions or chronic pain disorders, and had all successfully undergone a normal neurological examination. To exclude potential spectrum bias, we assessed the specificity of our algorithm using both healthy individuals and clinically well-characterized disease controls [14].

### MRI acquisition and image processing

A 3T Siemens Verio scanner was used for the neuroimaging of all patients using identical protocols. Three-dimensional data were acquired with a T_1_-weighted magnetization prepared rapid acquisition gradient echo (T_1_WI MPRAGE) sequence (repetition time [TR] = 2,300 ms, echo time [TE] = 2.53 ms, flip angle = 12°, field of view [FOV] = 256 × 256 mm, no gap, voxel size = 1.0 mm × 1.0 mm × 1.0 mm) and a T_2_-weighted fluid-attenuated inversion recovery (T_2_WI FLAIR) sequence (TR = 7,000 ms, TE = 80 ms, flip angle = 120°, FOV = 256 × 256 mm, no gap, voxel size = 1.5 mm × 1.5 mm × 1.5 mm).

Cortical reconstruction was achieved with the *FreeSurfer* (Version 6.0; https://surfer.nmr.mgh.harvard.edu) [15]. The reconstruction consisted of: (i) white matter (WM) segmentation; (ii) GM/WM boundary tessellation; (iii) inflation of the folded surface tessellation; (iv) automatic correction of topological defects; and (v) pial surface improvement using FLAIR data [16]. *FreeSurfer* parcellation was used for shape extraction, after which topological correction and mild smoothing were performed based upon a topology-preserving level set algorithm, as detailed previously [17, 18]. The ENIGMA-Epilepsy protocol was used for quality control analyses of these cortical reconstructions based upon the internal surface method (www.enigma.ini.usc.edu). Visual verification of surface extraction data was then performed, with manual correction of topological defects being conducted where necessary. Within-subject registration of FLAIR scans to T_1_-weighted images was performed. Extracted neocortical and subcortical surface-based features included:

(i) Cortical thickness was measured as the distance between the corresponding vertices of the GM/WM and pial This parameter is indicative of multiple cellular-level features such as size, density, neuronal arrangement, neuroglia, and nerve fibers [19].
(ii) Surface area was defined as the average surface area determined by 6 triangular meshes surrounding each vertex along the GM/WM interface, and is thought to reflect relative cortical column expansion or compression in a particular area [19].
(iii) Myelin levels were defined by the ratio of T_1_-weighted to T_2_-weighted signal intensity. This feature corresponded to the GM myelin content and the analysis was more reliable with in bias field removal, mapping of cortical GM voxels to the surface, and surface-constrained smoothing to reduce noise [20].
(iv) Radial distance corresponding to the distance of the vertex from the medial curve of the structure, enabling a shape thickness measure [21, 22].
(v) Jacobian determinant corresponding to the ratio of the area of an individual shape relative to the template area at the corresponding This parameter corresponds to localized tissue reduction or surface area enlargement relative to the corresponding template shape [21, 22].

All feature maps were registered to an average space (*fsaverage_sym*) with an identical number of vertices for each hemisphere. The right affected hemisphere vertex values of each feature were flipped to the left hemisphere following the *xhemi* (https://surfer.nmr.mgh.harvard.edu/fswiki/Xhemi) procedure [23], which is able to move all the feature maps to the left hemisphere to permit further intergroup comparisons.

### Feature selection and machine learning model development

The neocortex was parcellated at 180 regions per hemisphere using the HCP MMP 1.0 atlas [24], which delineates the cortical architecture, function, and connectivity. The subcortical structures were divided into 7 regions per hemisphere via a shape analysis approach, which primarily consisted of 4 steps: volume parcellation, surface extraction, registration, and local shape volume computation [25]. Initially, 568 candidate features were generated for classification. To avoid multicollinearity, a principal component analysis (PCA) was implemented to preserve 10 features for classifier training.

A SVM algorithm was employed for classification in the present study. This algorithm represents a hyperplane in multidimensional space, thereby minimizing error while allowing for maximal between-class separation. A linear kernel was chosen to contrast with the characteristic non-linear approach. The soft margin parameter that controls the trade-off between having zero training errors and allowing misclassifications was tuned using a possible range of values. A leave-one-out approach was used to cross-validate the classifier, with a given patient being classified according to the data from all other patients. The performance of the final model was assessed based upon the area under the curve (AUC) values for receiver operator characteristic (ROC) curves assessing individual and global features. Specificity was assessed based upon the proportion of healthy and disease control patients that were incorrectly identified as TN patients. Model robustness was assessed through 5,000 permutation tests. In addition to the SVM algorithm, we also investigated other ML approaches including logistic regression and ridge classifiers using 2-fold, 5-fold, 10-fold and leave-one-out (LOO) cross-validation, respectively (Table S1).

### Statistical analysis

Whole-brain morphological alterations, cortical thickness, surface area, myelin levels in the neocortex, and the radial distance and deformation of subcortical structures were statistically compared between TN patients and controls using surface-based linear models implemented in *SurfStat* (http://www.math.mcgill.ca/keith/surfstat/) through surface-wise Student’s *t*-test analysis. Models included age and sex as covariates. As the selected surface features are markers of TN-related brain alterations, Hotelling’s *t*-squared statistic was used to repeat the above comparisons based upon their multivariate combination in order to define the overall abnormality load. A diffusion kernel (full width at half maximum = 20 mm) that respects surface topology was used to blur surface-based measurements prior to analysis [19]. Random field theory was used to correct all statistical analyses, with family-wise error (FWE) being controlled for at P_FWE_ < 0.05.

Each parameter was assessed for the normality of distributed data using the Lilliefors test. Subgroup heterogeneity and dichotomous data were analyzed with Chi-squared variance tests. Normally distributed data were analyzed using Student’s *t*-tests, while Mann-Whitney *U* tests were used to compare nonparametric continuous variables. Correlation analyses were used to measure the relationships between variables, with Pearson correlation coefficient being employed for normally distributed data and Spearman’s rank correlation coefficient being conducted for non-parametric continuous variables. A *P* value < 0.05 was considered to be statistically significant.

## Results

### Participants

In total, this study enrolled 43 TN patients, 40 HFS disease control patients, and 43 healthy controls. All groups were age-matched (mean ± standard deviation [SD]: TN = 59.05 ± 9.95; HFS = 51.50 ± 9.04; controls = 58.95 ± 9.40; except analysis between TN and HFS, *P* < 0.05), sex-matched (female: 53.49 % TN, 60.00 % HFS, 55.81 % controls), and matched with respect to affected side (left: 48.84 % TN, 60.00 % HFS). The mean disease duration at time of MRI acquisition was 5.41 ± 4.71 years. Patients with TN reported pure V1 pain in 2.32 % of cases, V1 and V2 pain in 2.32 % of cases, V1, V2, and V3 pain in 6.98 % of cases, pure V2 pain in 27.91 % of cases, V2 and V3 pain in 23.26 % of cases, and pure V3 pain in 37.21 % of cases (Table 1).

**Table 1:** Patient demographic information

	TN	HFS	CON	Statistic
Numbers	43	40	43	/
Age, y	59.05 ± 9.95	51.50 ± 9.04	58.95 ± 9.40	TN vs. HFS: t = 3.61, *P* < 0.05*, MD = 2.09, 95 % CI = 3.38–11.71 TN vs. CON: t = 0.05, *P* = 0.97, MD = 0.09, 95 % CI = -4.06-4.24
Sex, female (%)	23 (53.49 %)	24 (60.00 %)	24 (55.81 %)	TN vs. HFS: Chi = 0.36, *P* = 0.55, OR = 0.77, 95 % CI = 0.32–1.83 TN vs. CON: Chi = 0.05, *P* = 0.83, OR = 0.91, 95 % CI = 0.39–2.13
Side, left (%)	21 (48.84 %)	24 (60.00 %)	/	TN vs. HFS: Chi = 1.04, *P* = 0.31, OR = 0.64, 95 % CI = 0.27–1.52
Duration, y	5.41 ± 4.71	/	/	/
Distribution of the pain (n, %)	V1 = 1 (2.32 %) V12 = 1 (2.32 %) V123 = 3 (6.98 %) V2 = 12 (27.91 %) V23 = 10 (23.26 %) V3 = 16 (37.21 %)	/	/	/

Continuous and categorical data were respectively analyzed via independent-samples *t*-test and Chi-squared variance test

Abbreviations: *TN* patients with trigeminal neuralgia, *HFS* disease controls with hemifacial spasm, *CON* healthy controls, *MD* mean difference, *95 % CI* 95 % confidence interval of the difference, *OR* odds ratio

The IRB of the Beijing Tiantan Hospital, Capital Medical University approved this study, with all participants having provided written informed consent to participate.

### Analysis of neocortical morphologic markers

**Fig. 1:**
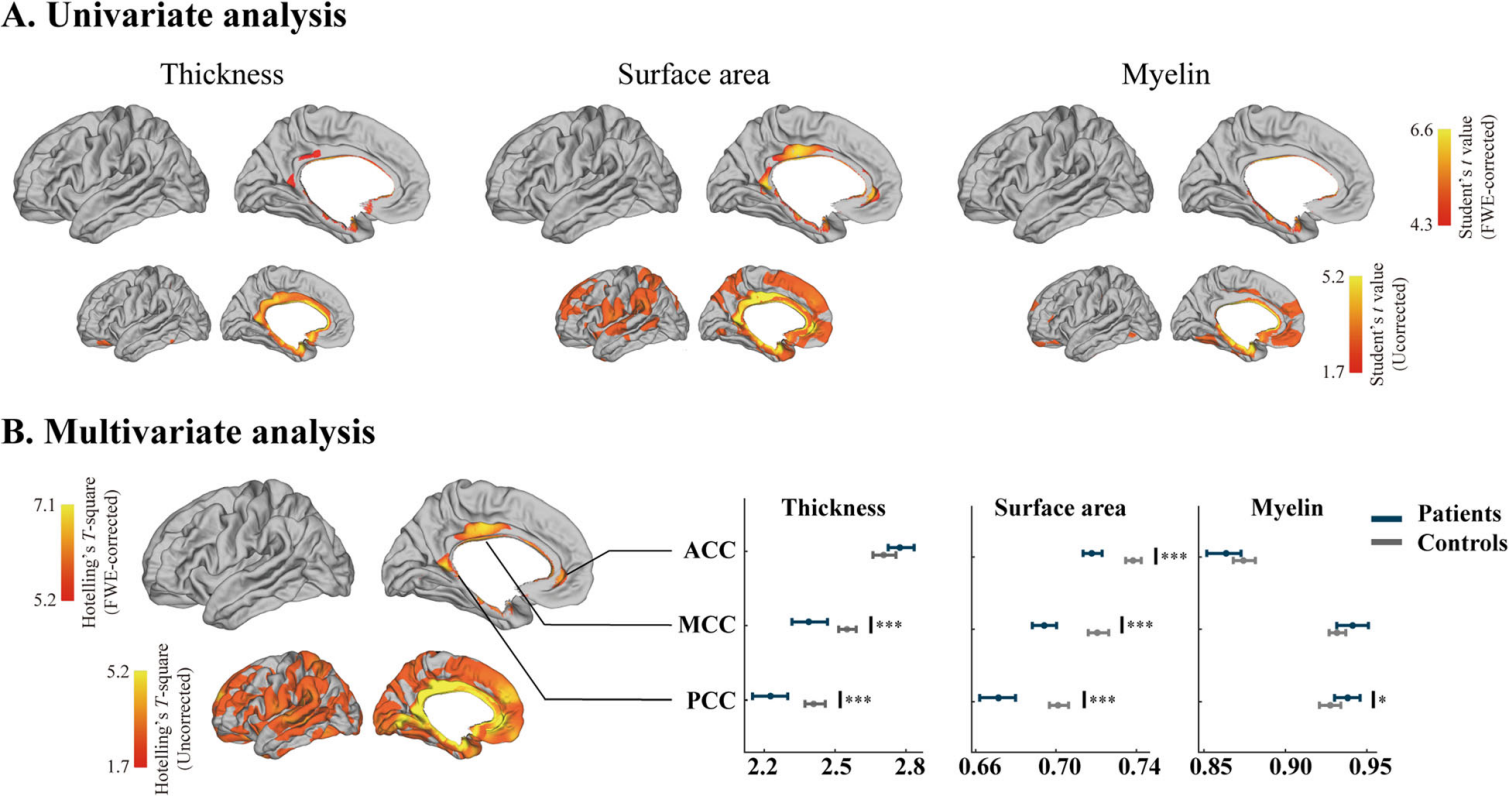
Neocortical surfaced-based feature analysis. **A** Univariate analysis identified clusters that differed significantly relative to controls. **B** Multivariate analysis of the joint distribution of thickness, surface area, and myelin. Comparisons between TN patients and controls with respect to the different neocortical features for different significant clusters. The *t* values are denoted by colored bars, with random field theory having been used to correct significant clusters for multiple comparisons at a P_FWE_ < 0.05. Abbreviations: ACC: anterior cingulate cortex; MCC: midcingulate cortex; PCC: posterior cingulate cortex; *: *P* < 0.05; ***: *P* < 0.001

Relative to healthy controls, TN patients exhibited cortical thinning in the midcingulate cortex (MCC) and the posterior cingulate cortex (PCC). TN patients also exhibited reduced surface area in the anterior cingulate cortex (ACC), MCC, and PCC, together with increased myelin levels in the entorhinal cortex and parahippocampal cortex (Fig. 1A). A surface-based, vertex-wise, FWE-corrected multivariate analysis indicated that TN patients exhibited significant differences between TN patients and controls in the ACC, MCC, and PCC. We further conducted ROI-wise statistical analysis of each morphologic feature in detail. Relative to controls, TN patients exhibited significant decreases in ACC surface area (Student’s *t* = -6.12, *P* < 0.001), cortical thickness (Student’s *t* = -3.68, *P* < 0.001), and MCC surface area (Mann-Whitney *U* = -4.95, *P* < 0.001), as well as decreased cortical thickness (Student’s *t* = -4.21, *P* < 0.001) and surface area (Student’s *t* = -6.12, *P* < 0.001), whereas myelin level in the PCC were increased (Mann-Whitney *U* = 2.39, *P* = 0.02) (Fig. 1B). Detailed statistical data corresponding to these three significant brain regions (ACC, MCC, and PCC) and surface-based features (cortical thickness, surface area, and myelin levels) are shown in Figure S1A. In areas corresponding to significant clusters, a multivariate linear model was used to directly evaluate the association between disease duration and individual surface-based features, with no differences being observed (Figure S1B). Furthermore, there were no significant correlations between duration and morphological features (cortical thickness: *R* = 0.13, *P* = 0.42; surface area: *R* = -0.09, *P* = 0.56; myelin: *R* = -0.10, *P* = 0.54; radial distance: *R* = -0.20, *P* = 0.20; Jacobian determinant: *R* = -0.25, *P* = 0.11) (Figure S2).

### Analysis of subcortical morphological markers

**Fig. 2:**
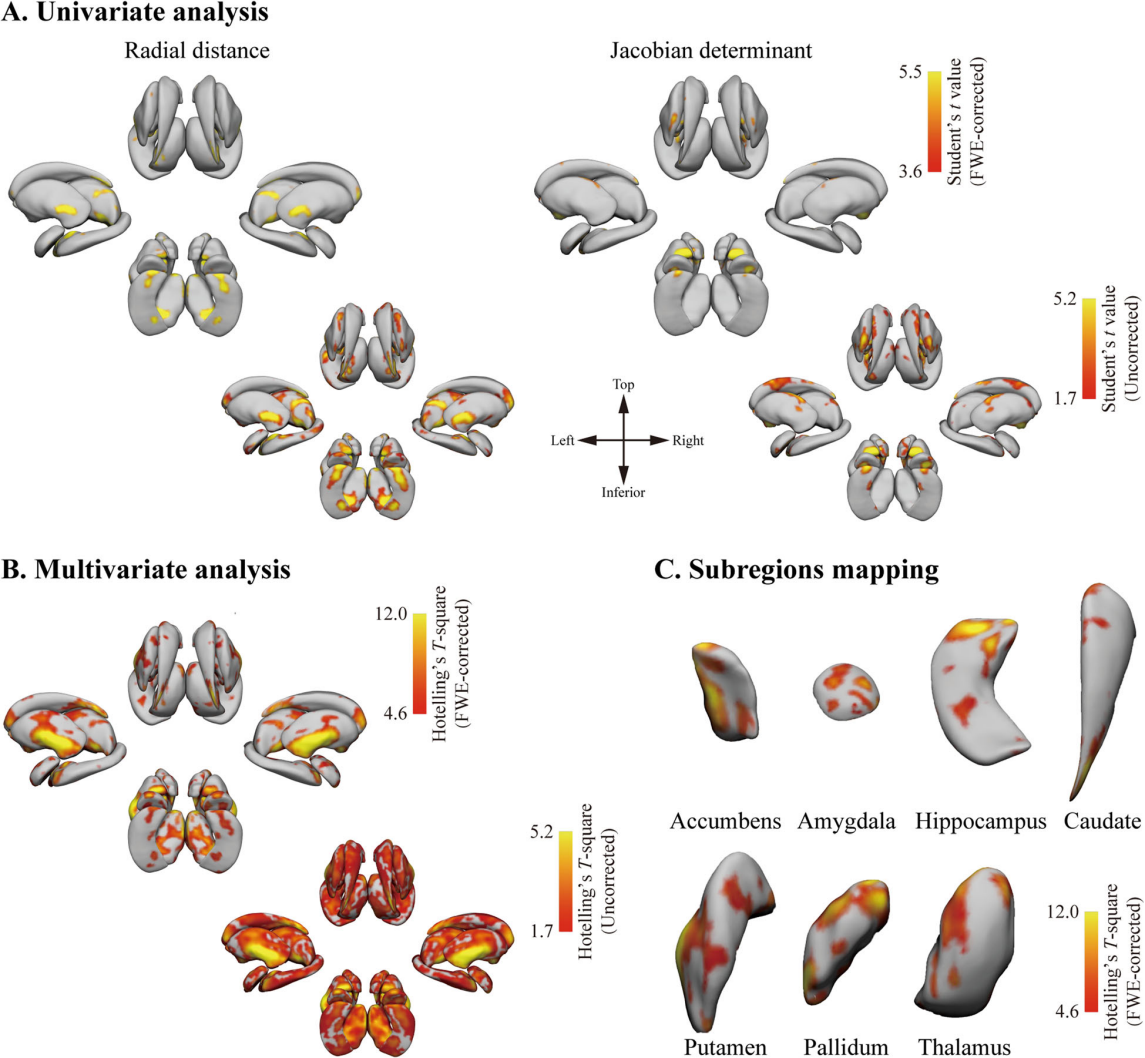
Subcortical surfaced-based feature analysis. **A** Univariate group analyses indicating clusters that were significantly altered relative to controls. **B** Multivariate analysis assessing the joint distribution of the radial distance and Jacobian deformation. Bar plots correspond to comparisons between patients and controls with respect to different subcortical features for different significant clusters. The *t* values are indicated by colored bars, with random field theory having been used to correct significant clusters for multiple comparisons at P_FWE_ < 0.05. **C** Subregional mapping results (left side only, as all samples were flipped such that the affected side was to the left)

TN patients exhibited a reduction in radial distance primarily in the head of the hippocampus, the lateral putamen, and the lateral thalamus, with Jacobian determinant reductions being evident in the ventromedial putamen relative to healthy controls (Fig. 2A). In an FWE-corrected multivariate analysis, the patient cohort exhibited widespread value reductions in the nucleus accumbens, the bilateral hippocampal head, the lateral putamen, and the ventral thalamus (Fig. 2B). Left subregion mapping results are also presented, as the affected side of all patients was flipped to the left (Fig. 2C).

### Automated patient classification

**Fig. 3:**
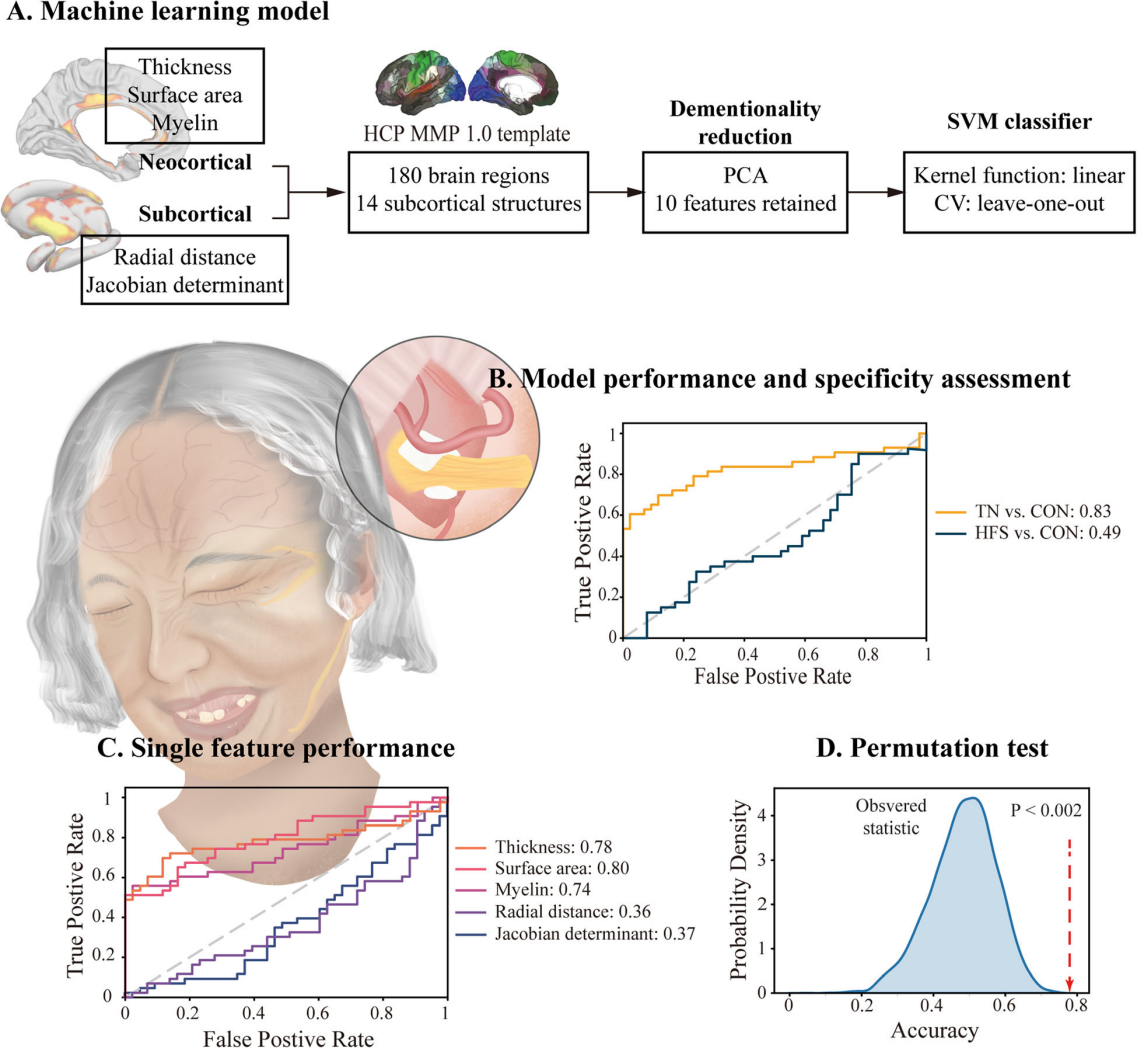
Machine learning model design and performance. **A** SVM classifier overview. HCP-MMP1.0: Human Connectome Project Multi-Modal Parcellation version 1.0 [24]; PCA: principal component analysis; CV: cross-validation. **B** Model performance as a tool for differentiating between TN patients and healthy controls. HFS patients served as disease controls for analyses of model specificity. TN: trigeminal neuralgia; HFS: hemifacial spasm disease controls; CON: healthy controls. **C** ROC and AUC values for models trained on individual neocortical and subcortical features. **D** AUC values corresponding to 5,000 permutation tests conducted using the final model. The AUC frequency across 5,000 tests is denoted with a blue histogram, while the real AUC without permutations is indicated by a vertical red line

The SVM model incorporating surfaced-based features correctly classified 26 out of 43 patients with TN (60.47 % sensitivity) and 41 of 43 healthy controls (95.35 % specificity), and reached a mean AUC of 0.83. To further assess the specificity of this model, the analysis was repeated for patients with HFS, where the model was only able to correctly detect 17 of 40 disease controls, with a mean AUC of 0.49 (Fig. 3B). In this context, the model performed best with a predictive AUC of 0.80 for the surface area followed by the remaining individual features: cortical thickness (AUC = 0.78), myelin (AUC = 0.74), Jacobian determinant (AUC = 0.37), and radial distance (AUC = 0.36) (Fig. 3C). Permutation tests (5,000) iterations confirmed that the accuracy of this model exceeded levels attributable to chance (*P* < 0.002) (Fig. 3D).

### Narrative synthesis of whole-brain neuroimaging patterns

**Fig. 4:**
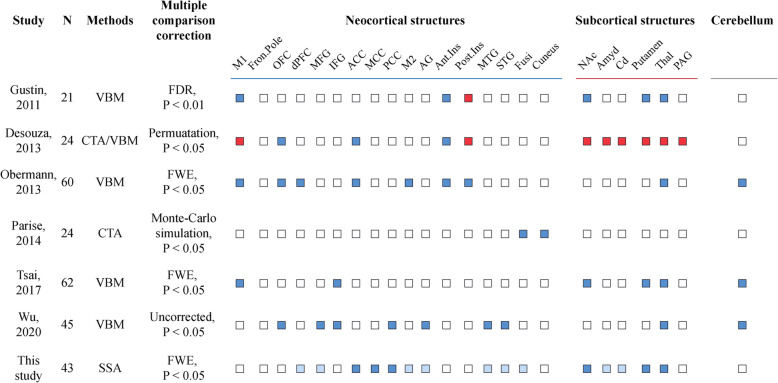
Systematic review of whole-brain studies assessing TN-related neuroimaging patterns. Sample size and quantitative MRI approaches are indicated along with reported findings (Red: increased cortical thickness or volume; Blue: decreased cortical thickness or volume, light blue: significant outcomes without correction; white: no assessment) across 3 brain subsystems (neocortical, subcortical structures and brainstem). VBM: voxel-based morphometry; CTA: cortical thickness analysis; SSA: surface-based shape analysis; FDR: false discovery rate; FWE: family-wise error; M1: primary somatosensory cortex; Fron. pole: frontal pole; OFC: orbitofrontal cortex; dPFC: dorsolateral prefrontal cortex; MFG: middle frontal gyrus; IFG: inferior frontal gyrus; ACC: anterior cingulate cortex; MCC: midcingulate cortex; PCC: posterior cingulate cortex; AG: angular gyrus; Ant.Ins: anterior insula; Post.Ins: posterior insula; Fusi: fusiform gyrus; Nac: nucleus accumbens; Amyg: amygdala; Cd: caudate nucleus; Thal: thalamus; PAG: periaqueductal gray

The majority of analyses evaluating TN-related neuroimaging patterns to date have focused on the neocortex, with several also having examined subcortical structures (6/7), while relatively few have assessed the brainstem (3/7) (Fig. 4). Approaches to evaluating whole-brain changes have included multi-region or voxel-based morphometry alone or in combination (*n* = 5), as well as cortical thickness analyses (*n* = 2). A moderate level of variation with respect to the statistical methods and thresholds employed has also been observed across studies. Progressive volume reduction was detected for frontal regions in 7/7 studies (100 %), parietal regions in 4/7 studies (57.1 %), temporal regions in 3/7 studies (42.9 %), the insular cortex in 2/7 studies (28.6 %), and subcortical regions in 6/7 studies (85.7 %), with the thalamus being reported in 6/6 studies (100 %) and the putamen in 4/6 studies (66.7 %). Cortical thickness and increased volume were also reported in 2/7 studies (28.6 %). Our TN cohort exhibited widespread abnormalities in multiple regions, although after correcting for multiple comparisons, significant morphological alterations were only detected in the ACC, MCC, PCC, and subcortical structures.

## Discussion

In the present study we examined whole-brain MRI morphological characteristics, including neocortical and subcortical alterations in patients with TN. In addition, we developed a two-step SVM machine learning approach that was employed to validate these TN-related morphological patterns. Participants cohorts did not differ with respect to demographics or affected side. Surface-based analyses revealed structural alterations in the ACC, MCC, PCC, and in multiple subcortical structures, particularly the lateral putamen and ventral thalamus. This brain prediction model yielded a satisfactory predictive AUC and specificity when sued to evaluate disease controls, and performed consistently in permutation tests, suggesting that these significant brain regions were potentially involved in TN-related brain network modulation.

### Morphologic alterations in neocortical structures

Neuroimaging studies conducted over the past decade have led researchers to characterize a so-called ‘pain matrix’ within the human brain composed of the thalamus, the basal ganglia, the supplementary motor cortex (SMC), the insular cortex, the ACC, and the prefrontal cortex (PFC) [26–28]. A better understanding of the mechanisms that lead to pain becoming chronic is necessary to guide the development of appropriate treatments capable of remediating inappropriate cortical reorganization.

Past neuroimaging studies have largely focused on structural changes associated with TN, such as GM reductions. For example, Gustine et al. reported a reduction in the GM volume in the primary somatosensory cortex, the anterior insula, the putamen, the nucleus accumbens, and the thalamus of patients with chronic orofacial pain, whereas GM volume in the posterior insula was increased [9]. Several other studies have similarly detected a reduction in GM volume in areas associated with pain processing, whereas GM volume is often found to be increased in other regions [28–30]. These reports, together with our results, strongly suggest a potential relationship between GM volume changes and altered pain perception or processing in TN patients.

We observed significantly decreased ACC, MCC, and PCC morphological indices in TN patients in this study. Several preclinical and clinical studies conducted to date have led investigators to define a “top-down” pain modulatory system in which pain perception can be increased or reduced by the activation of brain regions associated with modulating nociceptive input flow [31]. The ACC may play a role in pain adaptation, habituation, distraction, and the engagement of the endogenous pain control system [32]. The MCC has been linked to oriented attention, shifting attention, motor response, withdrawal reflex, motor control, and warning [33]. The PCC is thought to be associated with monitoring, consciousness, perspective-taking, and mindfulness [33].

### Morphologic alterations in subcortical structures

Subcortical structures are closely associated with pain processing, modulatory circuits, and related processes [34]. There is evidence that the putamen can mediate aspects of pain processing through sensorimotor/sensory-discriminative and reward/reinforcement networks [35]. The thalamus receives inputs from multiple ascending pain pathways, and is thus directly linked to the sensory discriminative and affective motivational aspects of pain. A study assessing neural oscillation revealed that local dynamic networks of neural oscillations in the thalamus participate in pain perception and modulation [36]. Owing to its subjective and complex nature, pain can also be influenced by emotional and cognitive factors and is not solely determined by nociceptive input. Given that the amygdala is important in the context of stress, anxiety, and emotional responses, it is likely to be an important site associated with the integration of these responses during pain processing [37]. The hippocampus is also integral to memory, mood, and stress-related processes [38]. The globus pallidus functions in the regulation of voluntary movement. Given the known roles of these structures, the amygdala, hippocampus, and globus pallidus are likely to play roles associated with pain-related negative emotional processing in TN patients [37].

### Neuroimaging features and statistical methods selection

Prior research has revealed that not all brain regions in the pain matrix are significantly altered in the context of TN, typically owing to variability with respect to sample size, image acquisition, postprocessing, and other methodological variables among studies [10]. The voxel-based morphometry (VBM) morphometric analysis program (MAP) is typically used as a post-processing approach to explore TN-related morphological alterations. While VBM methods are robust and can be readily implemented, they are subject to certain inherent limitations. They do not contain spatial relationships across the cortical surface, and registration errors may contribute to subtle changes being overlooked [39].

Herein, we report two primary findings. First, our results indicate that surface-based analysis processing approaches may be better suited to conducting quantitative, statistical morphometric analyses owing to their subvoxel accuracy and the potential for the detection of relatively subtle local changes. Second, rather than assessing only a single neuroimaging feature, we were able to extract data pertaining to cortical thickness, surface area, and myelin content in order to obtain a more comprehensive overview of TN-related morphological abnormalities. Together, these results may enable researchers to better establish the robustness of these findings, offering new insights into the nature of TN-related neuroanatomical changes [40].

### Limitation

The results of this study are subject to three primary limitations. For one, the sample size was relatively small owing to the exclusion of subjects when strict criteria were used, although this size was still larger than in previous studies. Second, no medication or psychological examination analyses were performed for study participants. In the future, assessment of mental status and medication use will be performed to further understand the psychopathological spectrum of TN. Finally, no patients with headache-related pain were included as disease controls, as high-resolution MRI data were unavailable for individuals with migraine or cluster headaches given that these conditions are not treated via a surgical approach. Future efforts to improve the underlying data will thus seek to overcome these limitations.

## Conclusions

In summary, these data demonstrate that TN is associated with distinctive whole-brain structural neuroimaging patterns, underscoring the value of machine learning as an approach to differentiating between morphological phenotypes, ultimately revealing the full spectrum of the disease, and highlighting relevant diagnostic biomarkers.

## Supplementary Information

**Additional file 1: Table S1.** Classification performance with different models and cross-validation strategies.

**Additional file 2: Figure S1. **Statistical analyses of neocortical features for different significant clusters. (A) Comparison of TN patients and controls. The t values (independence Student's *t-*test) or Z values (Mann-Whitney *U *test) are indicated with a colored bar. (B) Correlations between disease duration and neuroimaging values in TN patients. R values (Pearson correlation coefficients or Spearman's rank correlation coefficients) are indicated with a colored bar. *: *P* < 0.05; ***: *P* < 0.001.

**Additional file 3: Figure S2.** Relationship between duration and morphological features. No significant correlations were detected in the cortical thickness, surface area, myelin, radial distance, or Jacobian determinant. Grey dots indicate patients, the line indicates linear regression fit, and the band corresponds to the 95% confidence interval (CI). Spearman's rho correlation coefficients and *P* values are reported in each subplot.

## Data Availability

The datasets used and analyzed in the present study are available from the corresponding author on reasonable request.

## Abbreviations

ACC: Anterior cingulate cortex
AG: Angular gyrus
Amyg: Amygdala
Ant.Ins: Anterior insula
AUC: Area under the curve
Cd: Caudate nucleus
CTA: Cortical thickness analysis
dPFC: Dorsolateral prefrontal cortex
FDR: False discovery rate
FOV: Field of view
Fron. pole: Frontal pole
Fusi: Fusiform gyrus
FWE: Family-wise error
GM: Gray matter
HFS: Hemifacial spasm
IFG: Inferior frontal gyrus
M1: Primary somatosensory cortex
MAP: Morphometric analysis program
MCC: Midcingulate cortex
MFG: Middle frontal gyrus
MRI: Magnetic resonance imaging
Nac: Nucleus accumbens
OFC: Orbitofrontal cortex
PAG: Periaqueductal gray
PCC: Posterior cingulate cortex
Post.Ins: Posterior insula
REZ: Root entry zone
ROC: Receiver operator characteristic
SSA: Surface-based shape analysis
SVM: Support vector machine
T_1_WI MPRAGE: T_1_-weighted magnetization prepared rapid acquisition gradient echo
T_2_WI FLAIR: T_2_-weighted fluid-attenuated inversion recovery
TE: Echo time
Thal: Thalamus
TN: Trigeminal neuralgia
TR: Repetition time
VBM: Voxel-based morphometry
WM: White matter
