# Supplementary material for: Whole-brain morphological alterations associated with trigeminal neuralgia
Source: J Headache Pain. 2021 Aug 13;22(1):95. doi: 10.1186/s10194-021-01308-5 (PMC8362283; doi:10.1186/s10194-021-01308-5)
Supplement: Supplementary file 1 — Additional file 1: Table S1. Classification performance with different models and cross-validation strategies. [file 10194_2021_1308_MOESM1_ESM.docx]

Whole-brain morphological alterations associated with trigeminal neuralgia

**Table S1.** Classification performance with different model and cross-validation.

|  | **SVM** | **Logistics regression** | **Ridge classifier** |
| --- | --- | --- | --- |
| **2-Fold** | 0.75 | 0.73 | 0.69 |
| **5-Fold** | 0.80 | 0.80 | 0.79 |
| **10-Fold** | 0.82 | 0.81 | 0.75 |
| **LOOCV** | 0.83 | 0.80 | 0.74 |

SVM: support vector machine; LOOCV: leave-one-out cross-validation. Value is represented as the area under the curve (AUC).


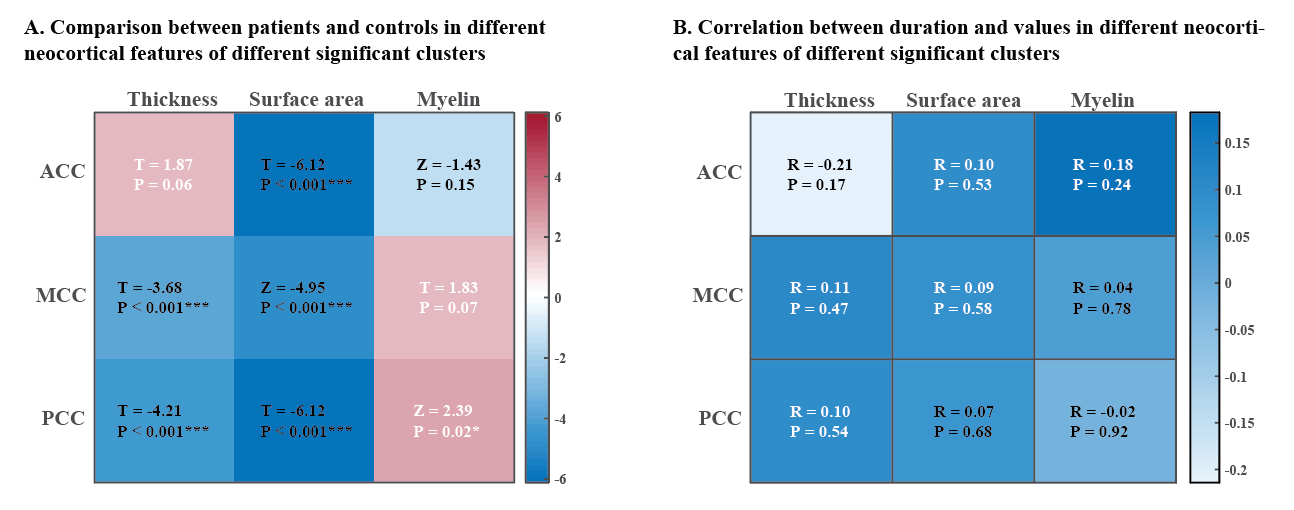


**Figure S1.** Statistical analyses of neocortical features for different significant clusters. (A) Comparison of TN patients and controls. The t values (independence student's *t-*test) or Z values (Mann-Whitney *U* test) are denoted with a colored bar. (B) Correlations between disease duration and neuroimaging values in TN patients. R values (Spearman rank correlation) are denoted with a colored bar. *: P < 0.05; ***: P < 0.001.

**Machine learning classification methods**

***Feature selection (Dimensionality reduction): Principal component analysis***

Principal component analysis (PCA) is a well-established unsupervised method for feature reduction in neuroimaging. PCA reduces dimensionality by geometrically projecting the data into lower dimensions called principal components (PCs), with the aim of finding the best summary of the data using a limited number of PCs. PCA uses an orthogonal transformation to convert a set of observations of possibly correlated features into a set of values of uncorrelated features (PC). PCs are then ranked according to explained variance in descending order. In the present investigation, PCA was implemented before the CV framework; dimensionality was reduced by 1) extracting the minimum number of principal components whilst retaining cumulative 90% of the variance from the data in the training set only, 2) projecting all morphological maps onto the resulting principal components and 3) using the resulting values for classification and 4) projecting the test data into the same components derived from the training set, and using the former for testing.

***Feature scaling: Standardization***

Standardization was performed by removing the mean and scaling to unit variance. This procedure was applied to each feature independently. Standardization is a common requirement for many machine learning methods, since algorithms might behave poorly if the individual features do not resemble normally distributed data. In addition, features with bigger scales might dominate the loss function of the training algorithms. To avoid “double dipping”, the statistics (mean and variance) were obtained using only the training set, and these same values were used in the standardization of test set. This procedure was already performed in features extraction.

***Machine learning: Support vector machine***

Support vector machine (SVM) is a supervised machine learning technique that maps the input data into a feature space using a set of similarity functions known as kernels. In this feature space, the model finds the optimal separating hyperplane by finding the largest margin of separation between the two classes within the training set. Once the hyperplane is determined, it can be used to predict the class of new unseen observations. In this study, a linear kernel was chosen to contrast with the characteristic non-linear approach. The soft margin (C) parameter, that controls the trade-off between having zero training errors and allowing misclassifications, was tuned from a possible range of values (2^-5^, 2^-3^, ..., 2^13^, 2^15^) using grid search, i.e., all possible values in each range were tested.

***Cross-validation: leave-one-out***

Cross validation is usually used to generalize the training process. Leave-one-out cross validation (LOOOCV) is *k*-fold cross validation taken to its logical extreme, with *k* equal to *n*, the number of data points in the set. That means that *n* separate times, the function approximator is trained on all the data except for one point and a prediction is made for that point. As before the average error is computed and used to evaluate the model. The evaluation given by leave-one-out cross validation error (LOO-XVE) is good, but at first pass it seems very expensive to compute. Fortunately, locally weighted learners can make LOO predictions just as easily as they make regular predictions. That means computing the LOO-XVE takes no more time than computing the residual error and it is a much better way to evaluate models.
