# Supplementary figures and images for: Whole-brain morphological alterations associated with trigeminal neuralgia
Source: J Headache Pain. 2021 Aug 13;22(1):95. doi: 10.1186/s10194-021-01308-5 (PMC8362283; doi:10.1186/s10194-021-01308-5)

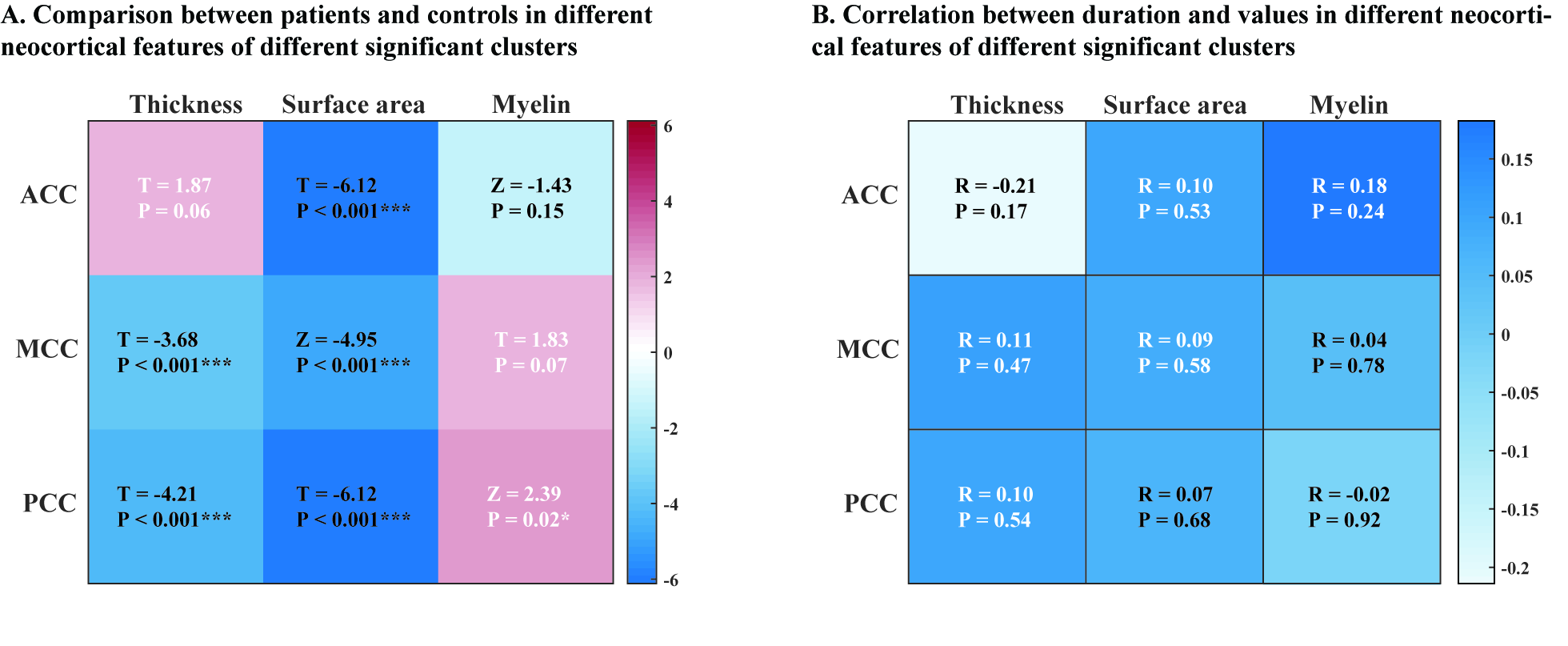

Supplement: Supplementary file 2 — Additional file 2: Figure S1. Statistical analyses of neocortical features for different significant clusters. (A) Comparison of TN patients and controls. The t values (independence Student's t-test) or Z values (Mann-Whitney U test) are indicated with a colored bar. (B) Correlations between disease duration and neuroimaging values in TN patients. R values (Pearson correlation coefficients or Spearman's rank correlation coefficients) are indicated with a colored bar. *: P < 0.05; ***: P < 0.001. [file 10194_2021_1308_MOESM2_ESM.tif]

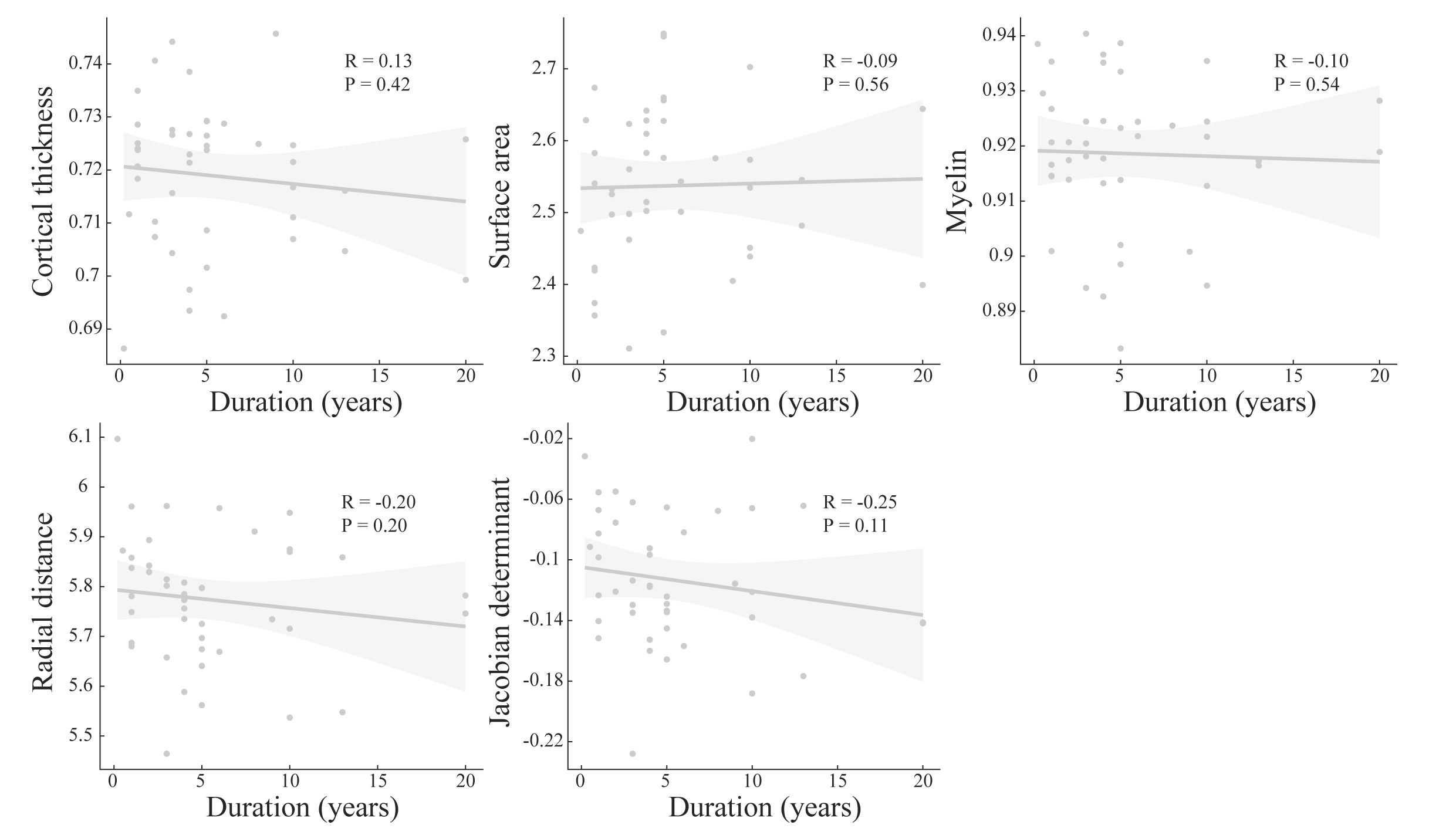

Supplement: Supplementary file 3 — Additional file 3: Figure S2. Relationship between duration and morphological features. No significant correlations were detected in the cortical thickness, surface area, myelin, radial distance, or Jacobian determinant. Grey dots indicate patients, the line indicates linear regression fit, and the band corresponds to the 95% confidence interval (CI). Spearman's rho correlation coefficients and P values are reported in each subplot. [file 10194_2021_1308_MOESM3_ESM.tif]
